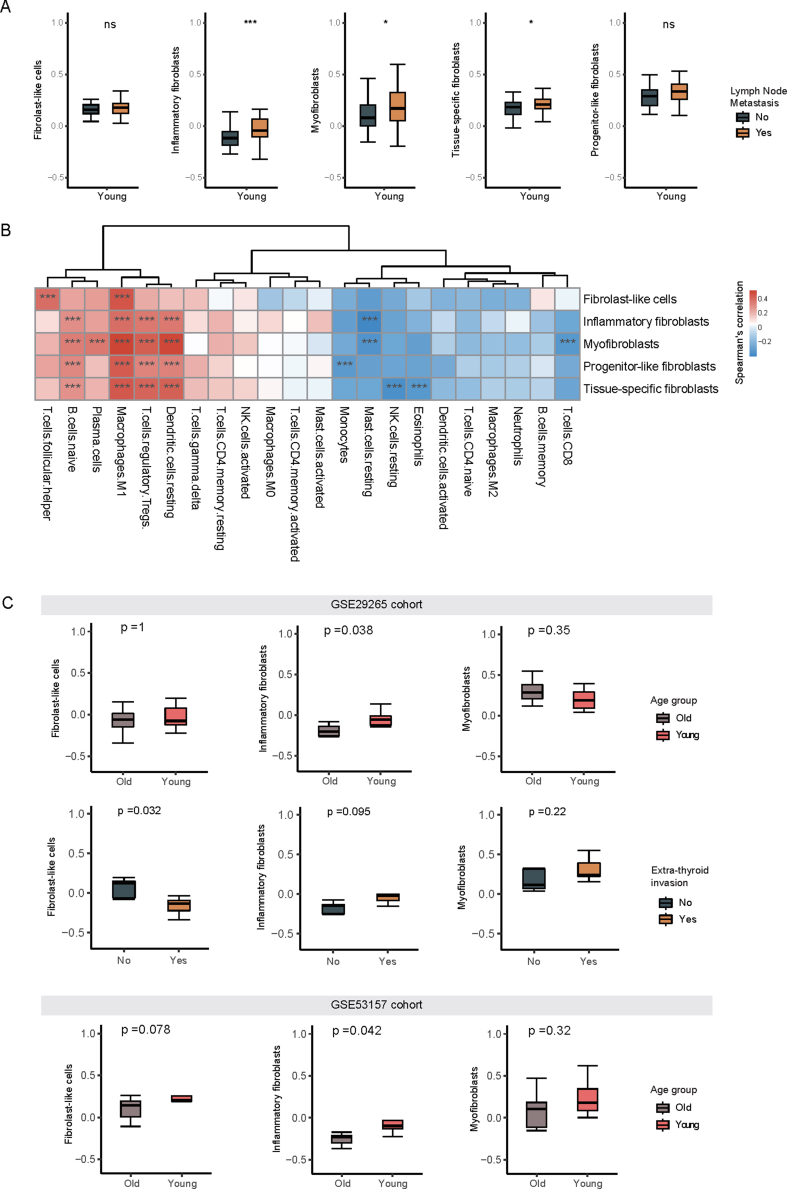# Integrative analysis reveals the clinical utility of cancer-associated fibroblast-derived signature, and its implication for young-onset thyroid cancer

**DOI:** 10.1016/j.gendis.2025.101882

**Published:** 2025-10-17

**Authors:** Shenglong Xu, Tianyao Chu, Hongyan Chen, Jing Wang, Yufei Wang, Yaoyao Liang, Xinlei Zhang, Yan Zhao, Peng Zhang

**Affiliations:** a Department of Otolaryngology, Head and Neck Surgery, Beijing Tongren Hospital, Capital Medical University, Beijing 100730, China; b Beijing Key Laboratory for Genetics of Birth Defects, Beijing Pediatric Research Institute, MOE Key Laboratory of Major Diseases in Children, Rare Disease Center, Beijing Children's Hospital, Capital Medical University, National Center for Children's Health, Beijing 100045, China; c Beijing ClouDNA Technology Co., Ltd., Beijing 101407, China; d Shanghai Institute of Immunology, Shanghai Jiao Tong University School of Medicine, Shanghai 200025, China

Thyroid cancer is the most common endocrine malignancy worldwide. Although prognosis is generally favorable, young patients show more advanced and aggressive features than adults, making prognosis prediction challenging.^1^ In solid tumors, cancer cells interact with the surrounding environment to form the heterogeneous tumor microenvironment (TME). Among TME components, cancer-associated fibroblasts (CAFs) are key producers of the extracellular matrix and regulators of the TME.^2^ Single-cell RNA sequencing has revealed diverse CAF phenotypes across cancer types, arising from distinct origins and activation states. This heterogeneity underscores the need to understand CAF diversity to identify tumor-specific biomarkers for guiding personalized treatment and follow-up strategies.^3^ However, pan-cancer research on CAFs is limited, with very few reports on thyroid cancer.^4^ This study first identified CAF clusters and signature genes using pan-cancer single-cell data. CAF composition and prognostic relevance were assessed across 23 solid tumors in the TCGA pan-cancer database. Finally, age-related CAF subtypes in thyroid cancer were analyzed.

A public single-cell dataset was utilized to determine signatures of different CAFs, including fibroblast-like cells (*MYH11* and *RGS5*), progenitor-like fibroblasts (*MFAP5* and *PI16*), myofibroblasts (*LRRC15*, *HOPX*, *MMP1*, and *SFRP2*), tissue-specific fibroblasts (*SOX6* and *ADAMDEC1*), and inflammatory fibroblasts (*IL6* and *HSPA6*) (Fig. 1A). These subtypes formed distinct clusters with unique gene expression patterns (Fig. 1B, top). Gene set enrichment analysis (GSEA) for genes specific to each fibroblast type on gene ontology biological process terms revealed enrichment in variant terms, except for progenitor-like fibroblasts (Fig. 1B, bottom). The top 15 differentially expressed genes of the five CAF subtypes were selected as signature gene sets (Table S1).

**Figure 1:**
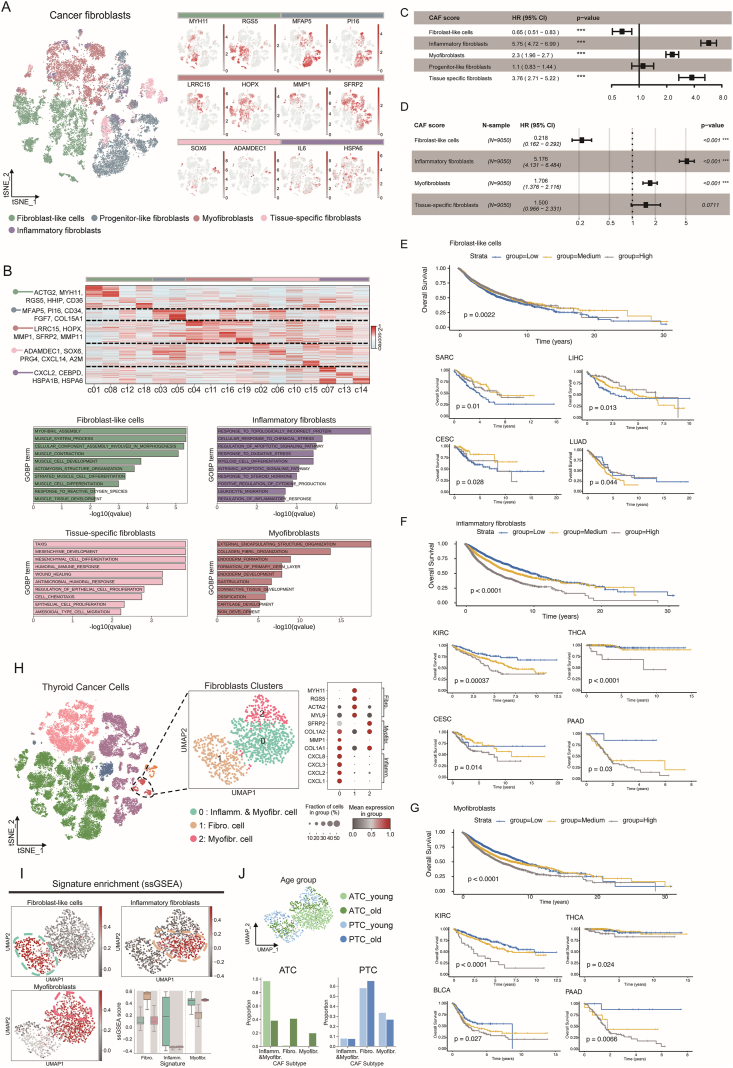
Age-related CAF subtypes in thyroid cancer correlate with clinical outcomes. **(A)** t-SNE visualization of five major cancer fibroblasts included in the GSE246219 dataset (left) and t-SNE visualization of expression of cancer fibroblast marker genes with a color bar on the top denoting the cancer fibroblast type (right). Cells were colored by cancer fibroblast type. **(B)** The heatmap showing the mean expression of the top 100 significantly highly expressed genes for each cluster (top), and the bar plot exhibiting enrichment results of significantly highly expressed genes for each major cancer fibroblast type on gene ontology biological process terms (bottom). The color bar at the top denotes the major fibroblast types. **(C)** Univariate Cox regression forest plot of CAF scores and overall survival (OS). **(D)** Multivariate Cox regression forest plot of CAF scores and OS. **(E)** Kaplan–Meier (K–M) curve for fibroblast-like cells score and OS at the pan-cancer level and in SARC, LIHC, CESC, and LUAD. **(F)** K–M curve for the inflammatory fibroblasts score and OS at the pan-cancer level and in KIRC, THCA, CESC, and PAAD. **(G)** K–M curve for myofibroblasts score and OS at the pan-cancer level and in KIRC, THCA, BLCA, and PAAD. **(H)** t-SNE visualization of eight cell types included in the GSE193581 dataset (left) and Uniform Manifold Approximation and Projection (UMAP) visualization of fibroblasts with expression of three cancer fibroblast marker genes (right). Cells were colored by cell type. **(I)** The UMAP plot exhibiting single-sample gene set enrichment analysis (ssGSEA) score of three cancer fibroblast signatures with statistical box plots on the lower right panel. Clusters with higher enrichment scores were highlighted. **(J)** The UMAP plot showing the distribution of cells from young- and old-onset patients with ATC and PTC (top) and the bar plots showing the proportions of three CAF subtypes in young- and old-onset patients for both ATC and PTC (bottom). Patients were stratified into young- and old-onset groups based on the median age at diagnosis for each histological subtype (PTC: 33 years; ATC: 59 years). CAF, cancer-associated fibroblast; SARC, sarcoma; LIHC, liver hepatocellular carcinoma; CESC, cervical squamous cell carcinoma and endocervical adenocarcinoma; LUAD, lung adenocarcinoma; PAAD, pancreatic adenocarcinoma; THCA, thyroid carcinoma; KIRC, kidney renal clear cell carcinoma; BLCA, bladder urothelial carcinoma; Fibro, fibroblast-like cells; Myofibr, myofibroblasts; Inflamm, inflammatory fibroblasts; ATC, adult trauma center; PTC, pediatric trauma center.

In the TCGA pan-cancer database, CAF subtype enrichment scores showed different patterns across cancer types, age groups, and genders (Fig. S1A). The relative abundances of immune cell subsets were compared between the high and low CAF score groups based on the median scores. CD8^+^ T cells, activated dendritic cells, and activated natural killer cells had higher immune enrichment in the low CAF score group, while plasma cells and M1 macrophages showed higher proportions in the high CAF score group (Fig. S1B). Univariate Cox regression analysis of CAF scores and overall survival revealed that inflammatory fibroblasts (hazard ratio/HR = 5.75; 95% confidence interval/CI: 4.72–6.99), myofibroblasts (HR = 2.3; 95% CI: 1.96–2.7), and tissue-specific fibroblasts (HR = 3.76; 95% CI: 2.71–5.22) were significantly associated with worse prognosis, while fibroblast-like cells (HR = 0.65; 95% CI: 0.51–0.83) were linked to better survival (Fig. 1C). Multivariate Cox analysis confirmed that inflammatory fibroblasts (HR = 5.176; 95% CI: 4.131–6.484) and myofibroblasts (HR = 1.706; 95% CI: 1.376–2.116) were associated with poorer prognosis, while fibroblast-like cells (HR = 0.218; 95% CI: 0.162–0.292) were linked to better survival (Fig. 1D). Kaplan–Meier curves and log-rank tests showed that CAF scores for fibroblast-like cells, inflammatory fibroblasts, and myofibroblasts were significantly associated with survival at both the pan-cancer level and in several individual cancers (Fig. 1E–G). Notably, grouping by inflammatory fibroblasts and myofibroblasts, CAF scores revealed consistent survival stratifications in pancreatic adenocarcinoma and thyroid carcinoma (THCA). The CAF scores differed significantly across groups based on distant metastasis, lymph node metastasis, and tumor staging (Fig. S2A–S2C), with specific fibroblast subtypes showing notable differences in lymph node metastasis status in several cancer types (Fig. S2D–S2G). In THCA, all four scores showed significant differences between metastatic and non-metastatic samples (*p* < 0.001).

To explore the relevance of CAF subtypes in early-onset disease, we analyzed samples from young-onset patients in the TCGA-THCA cohort. CAF scores for inflammatory fibroblasts, myofibroblasts, and tissue-specific fibroblasts were significantly elevated in cases with lymph node metastasis (Fig. S3A). Consistent with pan-cancer observations, Spearman correlation analysis revealed significant associations between multiple CAF scores and immune cell infiltration levels (Fig. S3B). Additionally, the inflammatory fibroblast score was significantly increased in the young-onset group within two independent external validation datasets (GSE29265 and GSE53157; *p* < 0.05). In dataset GSE29265, inflammatory fibroblast scores showed a trend toward increasing in samples with extrathyroidal invasion (*p* = 0.095), whereas fibroblast-like cell scores decreased significantly in this group (*p* = 0.032) (Fig. S3C).

We further examined the relationship between CAF subtypes and age using THCA single-cell sequencing data (GSE193581). Cells were clustered into subtypes, with fibroblasts divided into three subgroups: inflammatory & myofibroblasts (*CXCL8*, *CXCL3*, *CXCL2*, *CXCL1*, *SFRP2*, *COL1A2*, *MMP1*, *COL1A1*), fibroblast-like cells (*MYH11*, *RGS5*, *ACTA2*, *MYL9*), and myofibroblasts (*SFRP2*, *COL1A2*, *MMP1*, *COL1A1*) (Fig. 1H). The enrichment of CAF subtype signatures (fibroblast-like cells, inflammatory fibroblasts, and myofibroblasts) was assessed using single-sample GSEA, and the Uniform Manifold Approximation and Projection (UMAP) plots revealed distinct enrichment patterns for the three clusters (Fig. 1I). We examined the distribution of CAF subtypes across different age groups in both adult trauma center (ATC) and pediatric trauma center (PTC). In ATC, the young-onset group was predominantly composed of inflammatory & myofibroblasts, with significantly higher proportions than in the old-onset group, while the proportions of fibroblast-like cells and myofibroblasts were higher in the old-onset group. In PTC, fibroblast-like cells constituted over half of the CAF population in both age groups, with minimal differences observed between them (Fig. 1J).

In this study, we used single-cell transcriptomic analysis to characterize CAF heterogeneity, identifying distinct subtypes and signature genes. Each subgroup exhibited unique genes and functions. Notably, prior research identified two major CAF subtypes, “myCAF” (myofibroblasts) and “iCAF” (inflammatory fibroblasts).^2^^,^^3^ Our analysis confirmed the existence of these two subtypes. Myofibroblasts, characterized by the high expression of matrix metallopeptidase 1 (MMP1), MMP11, and leucine-rich repeat-containing 15 (LRRC15), play a crucial role in regulating extracellular matrix remodeling and modulating cell migration and invasion. In contrast, inflammatory fibroblasts are characterized by their immune-regulatory and pro-inflammatory roles, marked by high expression of C-X-C motif chemokine ligands (CXCLs) that promote immune cell recruitment, as well as elevated interleukin-6 (IL-6) levels. IL-6 from these fibroblasts has been shown to accelerate tumor cell proliferation, epithelial–mesenchymal transition, and metastasis.^3^^,^^5^ Integrating TCGA data, we confirmed the widespread presence of CAFs across cancer types at the pan-cancer level. Although CAF proportions vary among cancers, their consistent impact on immune infiltration and tumor prognosis suggests that CAFs play a significant role in cancer progression. Previous studies have reported the expression of CAF-associated proteins in the thyroid cancer stroma and found that the expression of certain CAF-related proteins is associated with lymph node metastasis and prognosis.^5^ Our study confirmed that inflammatory fibroblasts and myofibroblasts were significantly linked to survival and lymph node metastasis, with age-specific distribution observed in thyroid cancer.

Our study has several limitations. First, retrospective data from public databases may introduce selection bias. Future prospective and multicenter trials are needed to validate these findings, and the prognostic significance of CAFs for tumor treatment efficacy requires further evaluation. The functional roles and molecular mechanisms of genes in different CAF subtypes in thyroid cancer remain unclear and need further investigation. In addition, protein-level validation of key CAF markers was not performed in this study due to the lack of matched proteomic data, and future studies integrating single-cell transcriptomic and proteomic profiling are warranted.

In summary, our study revealed that CAFs serve as biomarkers for metastasis and prognosis at the pan-cancer level, with this role being particularly robust in thyroid tumors. This provides potential insights into the more advanced and aggressive characteristics of young-onset thyroid cancer. Further validation in larger cohorts is needed to confirm these results.

## CRediT authorship contribution statement

**Shenglong Xu:** Investigation, Formal analysis, Data curation. **Tianyao Chu:** Methodology, Formal analysis. **Hongyan Chen:** Investigation, Data curation. **Jing Wang:** Formal analysis. **Yufei Wang:** Investigation. **Yaoyao Liang:** Investigation. **Xinlei Zhang:** Supervision. **Yan Zhao:** Supervision. **Peng Zhang:** Writing – review & editing, Writing – original draft, Supervision.

## Data availability

The materials of patient cohorts used for the current study were publicly available and can be assessed by the TCGA database (https://portal.gdc.cancer.gov/). The single-cell RNA-sequencing datasets could be accessed from NCBI's Gene Expression Omnibus database (http://www.ncbi.nlm.nih.gov/geo/) through accession numbers: GSE246219 and GSE193581. The processed data and analysis codes are available upon reasonable request from the corresponding author.

## Ethics declaration

Ethics approval has been obtained from the ethics committee of Beijing Children's Hospital, Capital Medical University, for the use of data from the public database.

## Funding

This work was supported by the 10.13039/501100005090Beijing Nova Program (China) (No. Z211100002121044) and the Funding for the Reform and Development of the 10.13039/501100005088Beijing Municipal Health Commission (China).

## Conflict of interests

H.C., J.W., and X.Z. are employees of Beijing ClouDNA Co., and the other authors declare no competing financial interests.

## Figures and Tables

**figs1:**
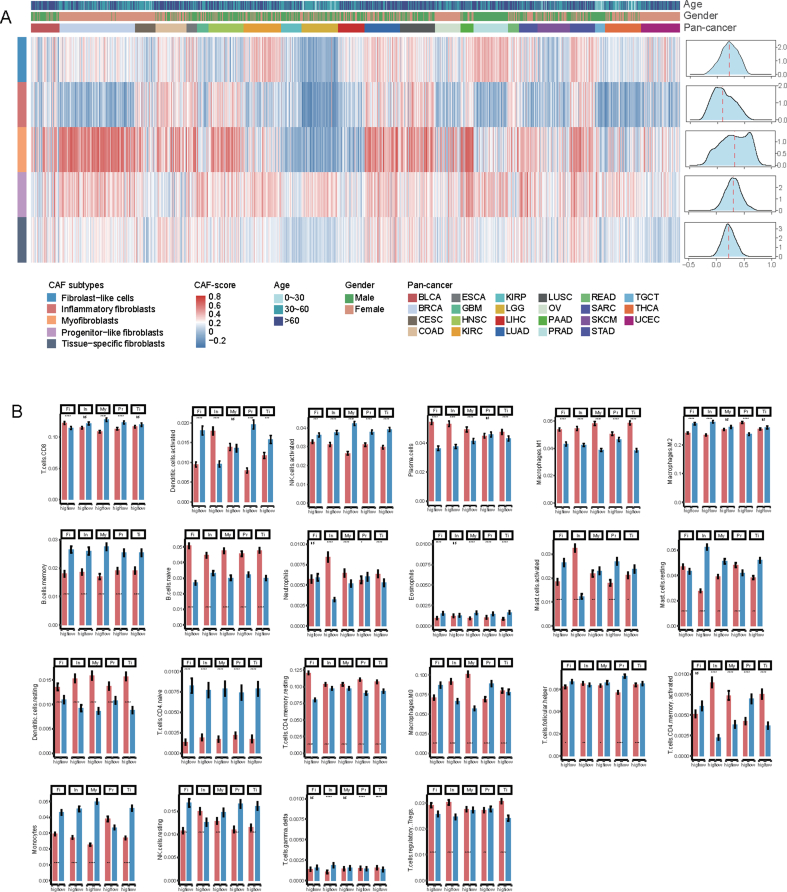


**figs2:**
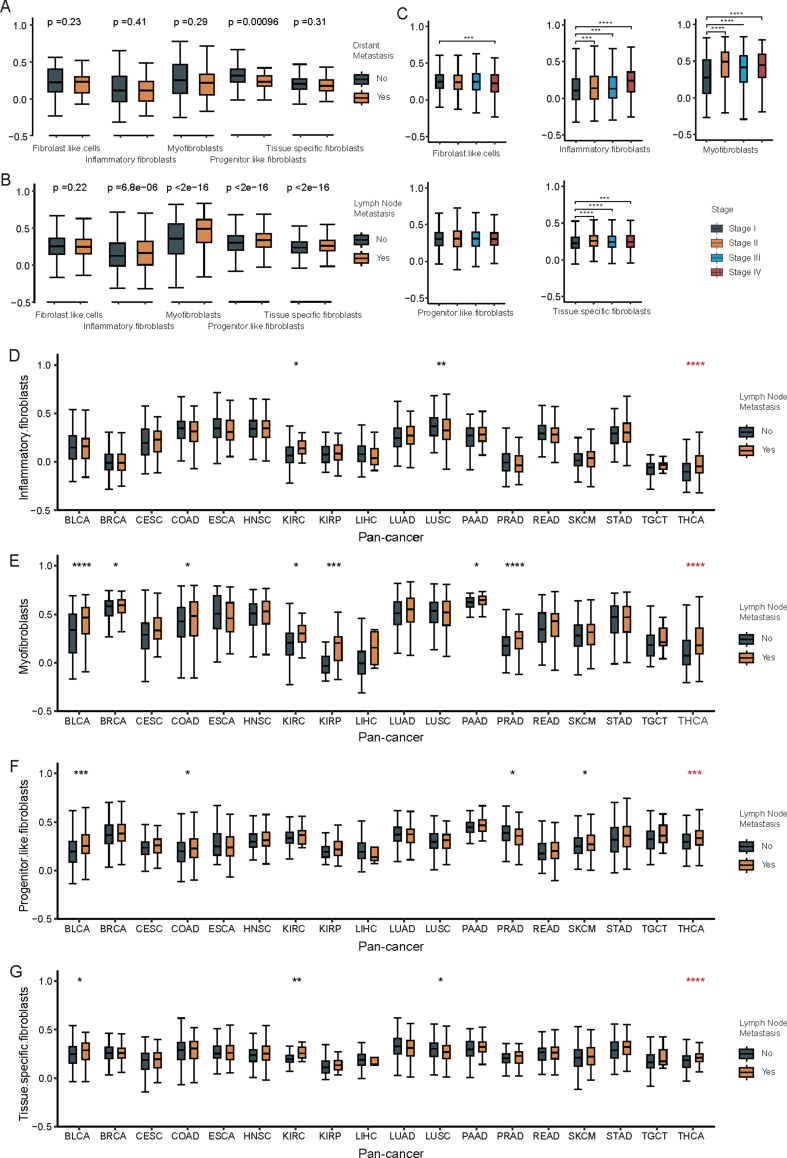


**figs3:**