# Supplementary material for: Integrative analysis reveals the clinical utility of cancer-associated fibroblast-derived signature, and its implication for young-onset thyroid cancer
Source: Genes Dis. 2025 Oct 17;13(5):101882. doi: 10.1016/j.gendis.2025.101882 (PMC13123491; doi:10.1016/j.gendis.2025.101882)
Supplement: Multimedia component 1 [file mmc1.docx]

**Supplementary Materials and Methods**

**Pan-cancer single-cell sequencing data analysis**

The single-cell RNA-seq (scRNA-seq) dataset was downloaded through the Gene Expression Omnibus (GEO) database (<https://www.ncbi.nlm.nih.gov/geo/>) under accession number GSE246219 [1]. Raw gene expression matrix and corresponding metadata were read and analyzed by Scanpy (v1.10.4) [2] in Python (v3.10). Cell labels were also obtained from the GSE246219 dataset, and we kept five major cancer fibroblasts (Fibroblast-like cells: 8289 cells, Progenitor-like fibroblasts: 7228 cells, Myofibroblasts: 6714 cells, Tissue-specific fibroblasts: 2462 cells, and Inflammatory fibroblasts: 1429 cells) for the next analysis. After normalizing the count matrix by the “*normalize_total”* and “*log1p”* functions, we used highly variable genes for subsequent analysis, which were calculated by the “*highly_variable_genes”* function. We employed principal component analysis (PCA) and BBKNN [3] for dimension reduction and batch effect correction, with “batch_key=‘SampleID’,n_pcs=30”. Then, t-distributed Stochastic Neighbor Embedding (t-SNE) was used for visualization. Finally, we obtained differentially expressed genes from the source study and performed Gene Set Enrichment Analysis (GSEA) on Gene Ontology Biological Process (GOBP) terms (<https://www.geneontology.org/>)[4] for each cancer fibroblast in R (v4.4.2) by the “*enricher*” function.

**TCGA patient cohort**

The Pan-Cancer Atlas (PanCanAtlas) initiative dataset [5] from The Cancer Genome Atlas (TCGA) Research Network includes RNA-seq expression profiles and clinical data for 33 distinct tumor types. The data were downloaded through the Xena database (https://xenabrowser.net/datapages/). After excluding non-solid tumors such as leukemia, pheochromocytoma, and paraganglioma, a total of 9,564 samples were included in the analysis. We further excluded tumor types with fewer than 120 samples, leaving 23 tumor types, which include a total of 9050 patients. In the TCGA-THCA dataset, samples were classified into two age groups: young-onset (≤35 years) and old-onset (>35 years).

**Construction of the CAF score**

In the TCGA Pan-cancer dataset, we calculated the CAF scores for each sample based on the top 15 signature genes of each CAF subtype identified in the single-cell analysis, excluding Mt-rRNA and IG_C_gene category genes *IGHA1*, *MT-RNR1*, and *MT-RNR2*. The enrichment scores of gene sets representing different CAF subtypes were calculated for each sample using the GSVA R package (Version 2.0.4). Patients were then classified into high/low groups or high/medium/low groups based on the median or quartiles.

**Evaluation of immune cells and differential analysis**

The proportions of 22 types of infiltrating immune cells in 9050 tumor samples were estimated using the CIBERSORT deconvolution method. The differences in cell proportions between different groups were calculated using the rank-sum test.

**External Validation Bulk RNA-Seq Datasets Acquisition and Processing**

The GSE29265 and GSE53157 [6] datasets were downloaded from the GEO database. GSE29265 contains data from 10 PTC samples, while GSE53157 includes 5 poorly differentiated thyroid carcinomas (PDTC), 7 PTC, and 4 follicular thyroid carcinomas (FTC). In both datasets, samples were classified into two age groups: young-onset (≤35 years) and old-onset (>35 years).

**Thyroid cancer single-cell sequencing data analysis**

The thyroid cancer scRNA-seq dataset was downloaded from the GEO database under accession number GSE193581[1] and preprocessed the data following the methods described above. This dataset includes 10 anaplastic thyroid cancer (ATC) tumors and 7 papillary thyroid cancer (PTC) tumors. The “Immune_All_High” model of celltypist (v.1.6.3) was used to predict cell type labels, and cellhint (v1.0.0) [7] with default parameters was applied to integrate cells from different samples. Then, we used t-NSE to visualize all cells included in the dataset. For re-clustering fibroblasts, we followed the pipeline of Scanpy mentioned above, and 10 PCs were used for dimension reduction and batch effect correction. Finally, three fibroblasts were clustered using the Leiden algorithm [8] with a resolution equal to 0.4. To annotate fibroblasts clusters, we examined the reported markers of three cancer fibroblast subtypes including Fibroblast-like cells (Fibro.) (*MYH11*, *RGS5*, *ACTA2*, and *MYL9*), Myofibroblasts (Myofibr.) (*SFRP2*, *COL1A2*, *MMP1*, and *COL1A1*), and Inflammatory fibroblasts (Inflamm.) (*CXCL8*, *CXCL3*, *CXCL2*, and *CXCL1*)[1]. Uniform Manifold Approximation and Projection (UMAP) was used for fibroblast cluster visualization. Additionally, we performed enrichment of the signatures of three cancer fibroblast subtypes for all fibroblasts using gseapy (v.1.1.3) [9], and compared the proportions of CAF subtypes between age groups (stratified by median age) across different pathological types.

**Statistical analysis**

Univariate and multivariate Cox regression analyses were performed using the survival R package (version 3.5-8), and 95% confidence intervals (CIs) and hazard ratios (HRs) were calculated. The log-rank test was applied to compare survival differences among the three patient groups to assess survival variations across different risk score groups. The differences in CAF scores across groups based on distant metastasis, lymph node metastasis, tumor staging, age groups, and extra-thyroid invasion status were compared using the rank-sum test. Spearman correlation tests were conducted to evaluate associations between CAF scores and the proportions of infiltrating immune cell types estimated by CIBERSORT. Statistical significance was set at p < 0.05.

**Supplementary Figures**

**Figure S1.**

**A.** Heatmap of CAF scores across 23 cancer types. **B.** Bar chart comparing immune cell infiltration between high and low CAF score groups. Significant differences are indicated as “*” for p < 0.05, “**” for p < 0.01, and “***” for p < 0.001.

**Figure S2.**

**A.** Boxplot comparing CAF scores between samples with and without distant metastasis at the pan-cancer level. **B.** Boxplot comparing CAF scores between samples with and without lymph node metastasis at the pan-cancer level. **C.** Boxplot comparing CAF scores across different clinical stages at the pan-cancer level. **D.** Boxplot comparing inflammatory fibroblast scores between samples with and without lymph node metastasis across different cancer types. **E.** Boxplot comparing myofibroblast scores between samples with and without lymph node metastasis across different cancer types. **F.** Boxplot comparing progenitor-like fibroblast scores between samples with and without lymph node metastasis across different cancer types. **G.** Boxplot comparing tissue-specific fibroblast scores between samples with and without lymph node metastasis across different cancer types. Significant differences are indicated as “*” for p < 0.05, “**” for p < 0.01, and “***” for p < 0.001.

**Figure S3.**

**A.** Comparison of CAF scores between young-onset TCGA-THCA samples with and without lymph node metastasis. **B.** Correlation heatmap illustrating relationships between CAF scores and infiltrating immune cell types in young-onset TCGA-THCA samples. Significant correlations are indicated as “*” for |cor| ≥ 0.3 and p < 0.05, “**” for p < 0.01, and “***” for p < 0.001. **C.** CAF score differences by age group and extrathyroidal invasion in the GSE29265 cohort (top) and CAF score differences by age group in the GSE53157 cohort (bottom).

**References**

1. Gao, Y., et al., Cross-tissue human fibroblast atlas reveals myofibroblast subtypes with distinct roles in immune modulation. Cancer Cell, 2024. 42(10).

2. Wolf, F.A., P. Angerer, and F.J. Theis, SCANPY: large-scale single-cell gene expression data analysis. Genome Biology, 2018. 19(1): p. 15.

3. Polański, K., et al., BBKNN: fast batch alignment of single cell transcriptomes. Bioinformatics (Oxford, England), 2020. 36(3): p. 964-965.

4. Thomas, P.D., et al., PANTHER: Making genome-scale phylogenetics accessible to all. Protein Science : a Publication of the Protein Society, 2022. 31(1).

5. Weinstein, J.N., et al., The Cancer Genome Atlas Pan-Cancer analysis project. Nature Genetics, 2013. 45(10): p. 1113-1120.

6. Pita, J.M., A. Banito, B.M. Cavaco, and V. Leite, Gene expression profiling associated with the progression to poorly differentiated thyroid carcinomas. British Journal of Cancer, 2009. 101(10): p. 1782–1791.

7. Xu, C., et al., Automatic cell-type harmonization and integration across Human Cell Atlas datasets. Cell, 2023. 186(26).

8. Traag, V.A., L. Waltman, and N.J. van Eck, From Louvain to Leiden: guaranteeing well-connected communities. Scientific Reports, 2019. 9(1): p. 5233.

9. Fang, Z., X. Liu, and G. Peltz, GSEApy: a comprehensive package for performing gene set enrichment analysis in Python. Bioinformatics (Oxford, England), 2023. 39(1).
